# Scientific Items

**Published:** 1884-09-20

**Authors:** 


### SCIENTIFIC ITEMS.

**Deep-Sea Curiosities.**—A very ingenious contrivance is the trawl which is ordinarily used for dredging the bottom of the sea. It may be sunk to a great depth, being attached to a steel wire rope four miles long, and is a canvas bag dragged on runners on the bottom, which keep the mouth of the bag in position to catch and hold the dredgings. On hauling the bag to the surface, the contents are poured into a great screen and carfully classified and preserved in alcohol. Some of the deposit from a half a mile or more below the surface was found under the microscope to be full of minute and beautiful shells. A fish found at six hundred fathoms was possessed of a body which was fully able to resist the pressure of a mountain of water upon it, estimated at three hundred pounds to the square inch, and, on being brought to the surface, the fish's eyes protruded from their sockets, and it was quite dead long before it reached the top of the water through the lack of resistance, which would burst an iron plate, but which was natural to the inhabitants of the great deep. The fish had eyes, though its home is supposed to be as dark as Erebus.—*Philadelphia Press.*

**About Salt.**—Common Salt, chemically known as chloride of sodium, is the most widely distributed substance in the body. It exists in every fluid and every solid; and not only is it everywhere present, but in almost every part it constitutes the larger portion of the ash when any tissue is burned. In particular it is a constant constituent of the blood, and it maintains in it a proportion that is almost wholely independent of the quantity that is consumed with the food. The blood will take up so much, and no more, however much we may take with our food; and, on the other hand, if none be given, the blood parts with its natural quantity slowly and reluctantly.

Under ordinary circumstances, a healthy man loses daily about twelve grains by one channel or the other, and, if he is to maintain his health, that quantity must be introduced. Salt is of immense importance in the process ministering to the nutrition of the body; for not only is it the chief salt in the gastric juice, and essential for the formation of bile, and may hence be reasonably regarded as of high value in digestion, but it is an important agent in promoting the processes of diffusion, and therefore of absorption. Direct experiment has shown that it promotes the decomposition of albumen in the body, acting, probably, by increasing the activity of the transmission of fluids from cell to cell. Nothing can demonstrate its value better than the fact that if albumen without salt is introduced into the intestines of an animal no portion of it is absorbed, while it all quickly disappears if salt be added.

If any further evidence were required, it would be found in the powerful instinct which impels animals to obtain salt. Buffaloes will travel for miles to reach a "salt-lick," and the value of salt in improving nutrition and the aspect of horses and cattle is well known to every farmer. The popular notion that the use of salt prevents the development of worms in the intestines has a foundation in fact; for salt is fatal to small thread-worms, and prevents their reproduction by improving the general tone, and the character of the secretion, of the alimentary canal. The conclusion, therefore, is obvious, that salt, being wholesome, and, indeed, necessary, should be taken in moderate quantities, and that abstention from it is likely to be injurious.—*Popular Science News.*

**Watery Vapor in the Air.**—M Montigny, of Brussels, has found that the presence of aqueous vapor in the atmosphere is always indicated by the preponderance of blue among the colors exhibited by a twinkling star, which is an infallible sign of rain. On the other hand, a deficiency of blue, and the preponderance of green, and also of violet, show that the air is in an abnormally dry state. On the strength of these indications, M. Montigny predicted that 1883 would be a dryer year in Brussels than those which had immediately preceded it, a prediction accurately fulfilled. Relying on the persistence of this same sign, he now announces that we have emerged from the period of wet years (which commenced in 1876), and may look forward to a series of finer and dryer ones. Meteorologists will regard this vaticination with considerable interest.—*Popular Science News.*

**Limits of Telephonic Action.**—The power of the telephone to transmit the voice to long distances is intimately associated with delicacy. Willoughby Smith has found by experience that a telephone will work through a "resistance" of wire corresponding to 150,000 miles of telegraph line; and hence it would seem mere child's play to fulfill the words of the poet, and "waft a sigh from Indus to the pole." But this was only a laboratory experiment; for on actual telegraph lines the leakage of electricity from the wire to the ground, damp and other drawbacks render the transmission of speech by wire far less easy in practice than was at first supposed. Nevertheless, it is on record that Mr. Edison transmitted speech over a line 750 miles long in America; and conversation has been carried on over 500 miles in India; 390 miles, from Tabrie, in Peru, to Tiflis; and 300 miles in Spain, Australia and other places where the atmostphere is dry and pure. In England, we have not been able to work through such long circuits, owing to the wetness of the atmostphere; but Mr. Van Rysselberghe, the ingenious chief of the meteorological observatory at Brussels, has telephoned from that city to Paris, a distance of 215 miles; and this while the same wire was carrying simultaneously an ordinary Morse telegraph message. By a peculiar disposition of his apparatus, Mr. Van Rysselberghe spoke to Paris by telephone without any interference from the Morse signals that were traversing the identical wire at the same time.—*Chambers' Journal.*

